# Antibiotics Use among Geriatric Patients Admitted in the Department of Medicine in a Tertiary Care Centre: A Descriptive Cross-sectional Study

**DOI:** 10.31729/jnma.8105

**Published:** 2023-06-30

**Authors:** Ruchi Shrestha, Brijesh Pandey, Sony Shakya Shrestha, Jyoti Tara Manandhar Shrestha, Pankaj Poudel

**Affiliations:** 1 Department of Pharmacology, Kathmandu University School of Medical Sciences, Dhulikhel, Kavrepalanchowk, Nepal; 2 Department of Cardiology, B.P. Koirala Institute of Health Sciences, Dharan, Sunsari, Nepal; 3 Department of Internal Medicine, Kathmandu University School of Medical Sciences, Dhulikhel, Kavrepalanchowk, Nepal

**Keywords:** *aged*, *antibiotics*, *drug utilization*

## Abstract

**Introduction:**

Ageing predisposes to increased risk of infections which make these population vulnerable to high risk of various chronic co-morbidities, organ dysfunction and mortality. Increased frequency of infections has led to an increasing proportion of geriatric patient admission to hospitals, and antibiotics therapy has long been recognized as a cornerstone in the treatment of infections. The aim of this study was to find out the prevalence of antibiotic use among geriatric patients admitted to the Department of Medicine in a tertiary care centre.

**Methods:**

A descriptive cross-sectional study was conducted among geriatric patients admitted to Department of Medicine in a tertiary care centre from 1 May 2022 to 31 August 2022. Ethical approval was obtained from the Institutional Review Committee (Reference number: 17/22). Patients with the age of ≥60 years, admitted to the Department of Medicine who stayed for at least 24 hours was included as the study population. Convenience sampling method was used. Point estimate and 95% Confidence Interval were calculated.

**Results:**

Among 520 geriatric patients, antibiotics was used in 252 (48.46%) (44.16-52.76, 95% Confidence Interval) patients. Ceftriaxone was the most common antibiotic used in 165 (65.48%) patients, followed by oral azithromycin in 72 (28.57%). The mean antibiotics used per patient was 1.59±0.73.

**Conclusions:**

The prevalence of antibiotic use in the geriatric population was found to be lower than in the other studies done in similar settings.

## INTRODUCTION

Infectious conditions predispose ageing patients to increased risk for morbidity and mortality.^1^ Incidence of infectious disease is higher in the older population because of the presence of chronic comorbidities, deteriorating organ functions and reduced host defence system.^2^

As the population ages, older adults make up an increasing proportion of patients admitted to hospitals, and antibiotics play an important role in the management of many diseases.^3^ Since the 2000s, there has been an increase of more than 20% in antibiotic prescriptions in elderly patients.^4^

The aim of this study was to find out the prevalence of antibiotic use among geriatric patients admitted to the Department of Medicine in a tertiary care centre.

## METHODS

A descriptive cross-sectional study was conducted in the Department of Medicine of Dhulikhel Hospital, Dhulikhel, Kavrepalanchowk, Nepal. Data from 20 December 2021 to 19 April 2022 were collected between 1 May 2022 to 31 August 2022 from the hospital records. Ethical approval was obtained from the Institutional Review Committee of Kathmandu University School of Medical Sciences, Dhulikhel Hospital, Kavre, Nepal (Reference number: 17/22). Patients with the age of >60 years, admitted to the Department of Medicine who stayed for at least 24 hours and hospital record with complete data were included as the study population. Those with incomplete hospital record data were excluded from the study. Convenience sampling method was done. The sample size was calculated using the following formula:


$$\begin{array}{ll} \text{n} & ={\text{Z}}^{2}\times \frac{\text{p}\times \text{q}}{{\text{e}}^{\text{2}}}\\ & =1.96^{2}\times \frac{0.50\times 0.50}{0.05^{2}}\\ & =385 \end{array}$$

Where,

n = minimum required sample size
Z = 1.96 at 95 % Confidence Interval (CI)
p = prevalence taken as 50% for maximum sample size calculation
q = 1-p
e = margin of error, 5%

The calculated sample size was 385. However, 520 samples were taken. Data was collected for demographics, co-morbid conditions, diagnosis, date of admission, date of discharge, antibiotics used and their dose, frequency, duration and route of administration. Data were collected from electronic medical records and case records and recorded in specially designed proforma.

Data were entered and analysed using IBM SPSS Statistics version 16.0. Point estimate and 95% CI were calculated.

## RESULTS

Among 520 geriatric patients admitted to Medicine Ward, antibiotics were used in 252 (48.46%) (44.1752.76, 95% CI) patients. The mean age of the patient was 71.38±8.09 years, the highest number of patients were from the age group 60-69 years 116 (46.03%). The male and female ratio was 1:1.02. Most patients admitted were from Kavrepalanchok district 153 (60.71%). The mean duration for hospital stay was 5.23±4.73 days, out of which 155 (61.51%) patients stayed for 1-5 days. Normal discharge was given in 215 (85.32%) patients (Table 1).

**Table 1:** Sociodemographic profile (n= 252).

Characteristics	n (%)
**Age (years)**	
60-69	116 (46.03)
70-79	92 (36.51)
80-89	41 (16.27)
90 and above	3 (1.19)
**Gender**	
Male	125 (49.60)
Female	127 (50.40)
**Address**	
Kavrepalanchok	153 (60.71)
Sindhupalchok	27 (10.71)
Sindhuli	22 (8.73)
Bhaktapur	13 (5.16)
Others	37 (14.68)
**Discharge type**	
Normal discharge	215 (85.32)
Discharge on request	26 (10.32)
Refer to other hospitals	5 (1.98)
Left against medical advice	6 (2.38)
**Hospital stay (days)**	
1-5	155 (61.51)
6-10	67 (26.59)
11-15	16 (6.35)
16-20	7 (2.78)
More than 20	7 (2.78)

**Figure 1:**
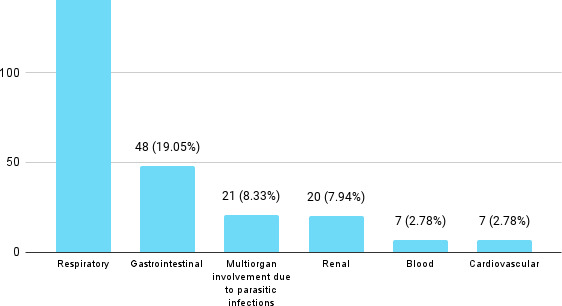
Commonly involved systems (n= 252).

Respiratory disease was the main cause for admission of 149 (59.13%) patients, for which they received antibiotics. Acute exacerbation of COPD (AECOPD) was the most common reason for receiving antibiotics, diagnosed in 102 (40.48%) patients. Co-morbidities were present in 157 (62.30%) patients, chronic obstructive pulmonary disorders (COPD) being the highest accounted co-morbidity in 89 (35.32 %), followed by hypertension 73 (28.97 %) patients (Figure 1).

The most common antibiotics used were from the cephalosporin group, given in 188 (74.6%) patients. Among all the antibiotics used, ceftriaxone was given in 165 (65.48%) patients, followed by azithromycin 72 (28.57%). The mean antibiotics used per person was 1.59±0.73 (Table 2).

**Table 2:** Antibiotics use (n= 252).

Antibiotics group	Antibiotics	n (%)
Cephalosporin	Ceftriaxone	165 (65.48)
	Cefixime	14 (5.55)
	Others (cefepime, cefotaxime, cefuroxime)	9 (3.57)
Macrolides	Azithromycin	72 (28.57)
	Others (clarithromycin, clindamycin)	3 (1.19)
Quinolones	Levofloxacin	24 (9.52)
	Others (moxifloxacin, ciprofloxacin)	10 (3.97)
Penicillin	Piperacillin with tazobactam	18 (7.14)
	Amoxicillin with clavulanic acid	6 (2.38)
	Others (amoxicillin, ampicillin+cloxacillin)	5 (1.98)
Tetracycline	Doxycycline	22 (8.73)
Carbapenems	Imipenem+cilastatin	10 (3.97)
	Meropenam	9 (3.57)
Amebicide	Metronidazole	10 (3.97)
	Others (secnidazole, metronidazole+ diloxanide furoate)	5 (1.98)
Others	Albendazole, linezolid, gentamycin, polymixin B, colistin, vancomycin, nitrofurantoin	23 (9.13)

Total antibiotics given in admitted patients was 419. One antibiotic was given in 136 (53.97%) patients, whereas four antibiotics were given in 5 (1.59%) patients (Table 3).

**Table 3:** Number of antibiotics used per person (n = 252).

**Number of antibiotics**	**n(%)**
One	136 (53.97)
Two	89 (35.32)
Three	22 (8.73)
Four	5 (1.59)

## DISCUSSION

In our study, the prevalence of antibiotic use in admitted geriatric patients was 252 (48.46%) which is lower than another study where the prevalence of antibiotic uses in admitted geriatric patients was 49.7% and 57.1% respectively.^5,6^ In our study, the mean age of the patient was 71.38±8.09 years. In the previous study, the mean age of the admitted patients was 78.37±7.9 years. This study showed the ratio of admitted females was higher than males, which was similar to the study in which 52.2% were female and 47.8% were male. The length of the hospital stay was 5.23±4.73 days. In a similar study, the length of hospital stay was 12.1±14.5 days.^7^

Among all the antibiotics used, the cephalosporin group was the most commonly used antibiotic, among which ceftriaxone was the most common one to be used. Ceftriaxone is a broad-spectrum, third-generation cephalosporin antibiotic.^8^ The study has concluded that dosage adjustment in ceftriaxone therapy is probably not necessary for elderly subjects, but in elderly patients who are debilitated, malnourished, or have marked impairment in renal function, the dosage of ceftriaxone may have to be reduced.^9^ In our study, azithromycin was the second most common antibiotic used for the study population. The pharmacokinetics and tolerability of azithromycin makes it particularly useful in the treatment of respiratory tract infections, sepsis, and enterocolitis.^10^ In the study conducted in India, cephalosporin was observed to be the most consumed antibiotic (33.2%), specifically cefotaxime (14.6%) and ceftriaxone (12.6%) in the admitted geriatric patients.^11^ Another study has shown ciprofloxacin as the most frequently prescribed antibiotic in admitted geriatric patients.^12^ Parenteral route was used for approximately two-thirds of the antibiotics. Increased parenteral use may be due to the fast onset of action that produce better outcome but also boost the cost of therapy, the risk of tissue injury and the transmission of blood-borne diseases.^13^

In our study, the average number of antibiotics used per person was 1.59±0.73. This data was higher compared to a previous study in which the average number of antibiotics prescribed per patient was 1.57.^11^ The mean number of drugs per prescription should be as low as possible because the higher the number of drugs, the greater the risk of drug resistance and high drug costs. In our study, about half of the patients were treated with one antibiotic, whereas the rest were treated with more than one antibiotic. The higher the number of drugs used concurrently, the higher the risk of occurrence of adverse effects, drug interactions, and medication errors.^14^

Our study has found the prevalence of more than half of the patients with respiratory conditions among admitted patients, among which AECOPD was most prevalent. In a study conducted in Nepal, lower respiratory tract infection was prevalent in 41.4% of the elderly population.^15^ In another study conducted among the elderly in Nepal, cancer followed by complications due to hypertension were shown to be the predominant reasons for admission in the geriatric population.^16^

This study has some limitations. The study was conducted in only one centre with a small sample size, hence the result cannot be generalized. Multicentric studies with large sample sizes are required to know the complete pattern of utilisation of antibiotics in geriatric inpatients.

## CONCLUSIONS

The prevalence of antibiotic use in the geriatric population was found to be lower than the other studies done in similar settings. The study highlights the trend of marginally higher utilisation of cephalosporins, especially third-generation in hospital settings. Strict antibiotic policy and antibiotic treatment guidelines are needed to be framed that enhance rational prescribing practices in geriatrics.
